# Optimized Extraction of Medicinal Mushroom Polysaccharides and Their Protective Effects Against 5-Fluorouracil-Induced Gastrointestinal Mucositis

**DOI:** 10.3390/ph19060946

**Published:** 2026-06-16

**Authors:** Jean Felipe dos Santos, Karien Sauruk da Silva, Marcello Iacomini, Fhernanda Ribeiro Smiderle, Daniele Maria-Ferreira

**Affiliations:** 1 Faculdades Pequeno Príncipe, Av. Iguaçu, 333, Rebouças, Curitiba CEP 80230-020, PR, Brazilfhernandas@gmail.com (F.R.S.); 2 Instituto de Pesquisa Pelé Pequeno Príncipe, Av. Silva Jardim, 1632, Água Verde, Curitiba CEP 80250-060, PR, Brazil; 3 Departamento de Bioquímica e Biologia Molecular, Universidade Federal do Paraná, Curitiba CEP 81531-980, PR, Brazil

**Keywords:** *Ganoderma lucidum*, polysaccharides, β-glucans, response surface methodology, intestinal mucositis, 5-fluorouracil, anti-inflammatory activity

## Abstract

**Background**: *Ganoderma lucidum* is a medicinal mushroom widely recognized for its high content of bioactive polysaccharides, particularly β-glucans with immunomodulatory properties. This study aimed to optimize polysaccharide extraction conditions to maximize yield and glucan content, and to evaluate the biological activity of the obtained fractions in an experimental model of intestinal mucositis. **Methods**: Polysaccharides were extracted using a combination of hot-water extraction and ethanol precipitation, optimized by response surface methodology. Optimal conditions (121 °C for 120 min followed by 90% ethanol precipitation) yielded a crude polysaccharide fraction (Poli-GL). A subsequent freeze–thaw process generated a soluble fraction (S-Poli-GL). Structural and compositional characterization was performed using enzymatic assays, monosaccharide profiling, and NMR spectroscopy. The biological effects of Poli-GL and S-Poli-GL were evaluated in a 5-fluorouracil-induced intestinal mucositis model following oral administration at doses of 30, 100, and 300 mg/kg. **Results**: The optimized extraction protocol enabled efficient recovery of polysaccharides enriched in glucans. S-Poli-GL exhibited a high total glucan content, including 43.3% β-glucans and 3.45% α-glucans, along with minor amounts of galactose and mannose. Structural analysis confirmed the predominance of branched β-(1→3),(1→6)-D-glucans. While Poli-GL did not prevent mucositis development, S-Poli-GL significantly reduced the disease activity index and attenuated intestinal inflammation, indicating enhanced biological activity associated with the soluble glucan-rich fraction. **Conclusions**: Optimization of extraction and fractionation improves the functional properties of *G. lucidum* polysaccharides. The soluble glucan-enriched fraction (S-Poli-GL) demonstrated significant protective effects in intestinal mucositis, supporting its potential as a therapeutic candidate and warranting further investigation for clinical application.

## 1. Introduction

Fungi are among the most diverse and abundant groups of organisms worldwide (Zotti et al., 2013) [1]. Mushrooms, a valued part of this kingdom, are often called “forest meat” because of their nutritional richness, offering a non-animal alternative with comparable nutritional value (Dimitrijevic et al., 2018) [2]. For thousands of years, mushrooms have been consumed for their therapeutic potential and used in traditional medicine (Wasser, 2011) [3].

*Ganoderma lucidum* (reishi) is notable among these therapeutically significant mushrooms. It is generally classified as a medicinal mushroom because its fruiting bodies contain active ingredients that positively affect human health. This species is a particularly rich source of bioactive compounds, including those extracted from its fruiting bodies, mycelium, and spores (Baby et al., 2015; Siwulski et al., 2015) [4,5]. Among these bioactive compounds are several different polysaccharides, including β-D-glucans, α-D-glucans, and triterpenes (Ahmad, 2018a) [6]. Their bioactive effects are attributed to their anti-inflammatory (Joseph et al., 2011) [7], immunomodulatory (P. Y. Wang et al., 2012) [8], and anti-cancer activities (Kao et al., 2016) [9].

A significant part of the biological activity of fungi is attributed to their complex polysaccharide composition. These polysaccharides, rich in mannose, galactose, and/or glucose, function both as storage molecules and as structural components of the fungal cell wall (Smiderle et al., 2017a) [10]. Structural polysaccharides include glucans, which are usually branched (1→3)(1→6)-β-glucans, although linear α-glucans and β-glucans have also been identified in several fungal species (Ruthes et al., 2015) [11]. In this context, structural variations such as branching patterns can directly affect physicochemical properties like solubility. The solubility of extracted β-glucan fractions may be related to their degree of branching: highly branched structures exhibit greater conformational flexibility in aqueous solution, which favors their solubility (Moreno et al., 2016) [12], whereas linear glucans have lower solubility in water.

Research has shown that mushroom-derived polysaccharides exhibit a range of bioactivities, including anti-inflammatory (Zhang et al., 2019) [13], immunomodulatory (Ahmad, 2018b) [6], antimicrobial (Corrêa et al., 2016) [14], antitumor (Fang et al., 2022) [15], and potential prebiotic effects (Ruthes et al., 2021) [16]. The therapeutic versatility of polysaccharides is largely attributed to their efficacy combined with a low incidence of side effects, making them a useful broad-spectrum therapeutic approach.

Intestinal mucositis is a major clinical challenge that requires new therapeutic interventions. It is characterized by marked inflammation and structural damage to the intestinal mucosa and often occurs as a significant adverse effect of cancer chemotherapy. 5-Fluorouracil (5-FU), a commonly used chemotherapeutic agent, is a major trigger of intestinal mucositis, leading to significant patient morbidity and negatively impacting treatment outcomes (Longley et al., 2003) [17]. Recent studies have shown that polysaccharides represent a promising therapeutic strategy to attenuate 5-FU-induced intestinal mucositis, as they can modulate inflammation and protect the intestinal epithelium (Sauruk da Silva et al., 2021) [18].

In addition, there is evidence that the polysaccharides of *G. lucidum* can improve the survival and activity of the gut microbiota, supporting their therapeutic potential for treating intestinal mucosal inflammation (Xie et al., 2019) [19].

Efficient extraction and purification of fungal polysaccharides is crucial for maximizing their therapeutic efficacy. Various extraction techniques have been used, including ultrasound-assisted extraction (UAE) (Alzorqi et al., 2017) [20], microwave-assisted extraction (MAE) (Smiderle et al., 2017b) [10], enzyme-assisted extraction (EAE) (Li & Wang, 2016) [21], pulsed electric field-assisted extraction (PEFAE) (Xue & Farid, 2015) [22], and hot water extraction (HWE) (Morales et al., 2019) [23]. While conventional methods such as hot water extraction (HWE) are widely used (Rodríguez-Seoane et al., 2018) [24], optimization of this technique is essential.

Ethanol precipitation, typically using concentrations of 50% to 90%, is a widely used technique for the pretreatment of polysaccharide extracts. Although often employed for the removal of lipids and pigments, its main application in polysaccharide isolation is as an initial step in precipitating crude polysaccharides from aqueous extracts. The effectiveness of this method relies on the different solubility of polysaccharides, enabling their selective precipitation according to molecular size and yield at various ethanol concentrations (Hu et al., 2019; Xu et al., 2014) [25,26]. Different ethanol concentrations for precipitating polysaccharides can result in different yields or structures (Hu et al., 2019; Xu et al., 2014) [25,26].

In this study, a response surface methodology (RSM)-based experimental design was used to determine the optimal conditions for obtaining *Ganoderma lucidum* polysaccharide extracts with high yield and enhanced bioactivity, and to investigate their effects in a 5-fluorouracil (5-FU)-induced intestinal mucositis animal model. Specifically, we showed that a simple post-extraction step produced a soluble fraction enriched in β-(1→3),(1→6)-glucans with enhanced biological activity, whereas the crude extract showed no protective effect. To the best of our knowledge, this is the first study to evaluate *Ganoderma lucidum* polysaccharides in a 5-FU-induced intestinal mucositis model.

## 2. Results and Discussion

### 2.1. Experimental Design and Optimization of Extraction Conditions

To determine the optimal conditions for maximizing β-glucan yield, a full factorial 32 design was developed using Statgraphics Centurion XVI 16.1.15 (Table 1). Extraction with hot water and pressure is the most used and cost-effective method for extracting polysaccharides from fungi. Therefore, an autoclave was used as the extraction device, and the extraction duration was defined as one of the independent factors. The levels were set between 20 and 120 min to ensure maximum extraction while preventing thermal degradation of the polysaccharide chains, which is often observed under prolonged hydrothermal treatments (121 °C, 1.2 atm) (Zavadinack et al., 2025) [27]. In addition, some studies observed that different concentrations of ethanol used to precipitate polysaccharides may affect the yield and composition of polysaccharides (Xu et al., 2014) [26]. This information guided the choice of the other independent factor: the concentration of ethanol used to precipitate polysaccharide, which was set from 30% to 90%, representing the practical and selective limit for effective polymer recovery. The experimental design followed the conditions set by the software, which generated eleven randomized extraction conditions (Table 1). Data were obtained from triplicate experiments for each condition, and extractions were performed in the order generated by the software. The reactions analyzed were total polysaccharide, measured as dried mass (mg), and total glucose content (%, as an indirect measurement of glucans) (Table 1).

**Figure 1:**
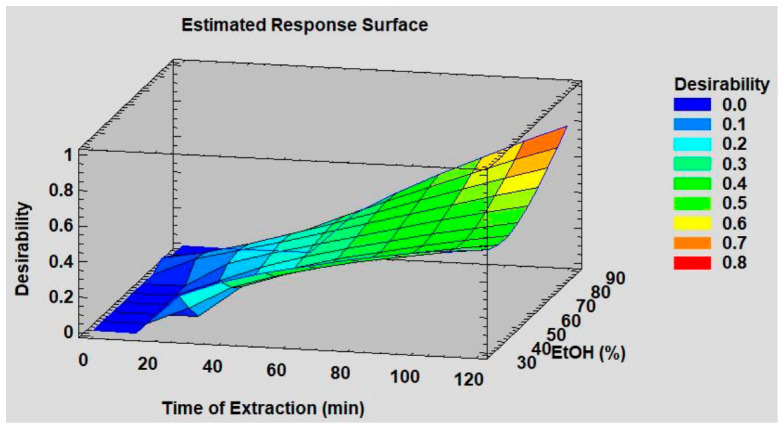
3D response surface plot (desirability function) of extracts from *G. lucidum*. Variable responses analyzed: polysaccharide yield (mg) and glucose content (%).

The extraction conditions that resulted in the highest yield and glucose content were 120 min of autoclave extraction and 90% ethanol for polysaccharide precipitation. Under these conditions, a polysaccharide yield of 1.5% (15 mg per 1 g of mushroom) and a glucose content of 18.2% were obtained. Data were calculated based on the overall mean yield and glucose concentration for each extraction condition, which were performed in triplicate, and then used to create the graphical representation. The reaction plot shown in Figure 1 clearly demonstrates that as extraction time increases, both yield and glucose concentration increase, indicating that no degradation occurs during the process. The same pattern was observed with increasing concentrations of ethanol used to precipitate the polysaccharides.

The most widely used method for achieving high polysaccharide yields at lower extraction costs is hot water extraction (HWE) (W. Wang et al., 2022) [28]. This method, commonly used by various researchers, utilizes water at temperatures between 50 °C and 100 °C, with extraction times typically ranging from 1 to 5 h. Several studies have demonstrated the effectiveness of HWE for extracting polysaccharides (Morales et al., 2019) [23].

The monosaccharide composition of each extract prepared by the RSM method was analyzed, and the main monosaccharides detected were glucose, galactose, and mannose, with traces of fucose and arabinose. The relative amount of glucose compared to the other monosaccharides exceeded 88% in all fractions (Table 2).

Once the optimal conditions were established, a medium-scale extraction was performed with 50 g of mushrooms extracted at 121 °C and 1 atm for 120 min. The extract was concentrated under reduced pressure, and ethanol was added until a concentration of 90% was reached. The precipitated polysaccharides were recovered by centrifugation and freeze-drying. Medium-scale extraction was performed in two separate batches, and the mean yield was 0.86 g ± 0.01 g (1.72%). Subsequently, both batches were combined, and a purification procedure was carried out to separate the soluble from the insoluble polysaccharides. For this purpose, the polysaccharide solution was frozen, then thawed, and centrifuged (10,000 rpm, 20 min, at 4 °C). This procedure yielded two fractions: S-Poli-GL (0.4 g) and P-Poli-GL (1.16 g). In this study, only the soluble fraction (S-Poli-GL) was used, which contained the following monosaccharides: glucose (85.0%), galactose (8.6%), and mannose (6.7%). The presence of mannose and galactose is consistent with other studies.

Glucans are the main polysaccharides in the fungal cell wall, and their content in the S-Poli-GL fraction was determined using an enzymatic method (K-YBGL, Megazyme, Wicklow, Ireland) that quantifies total, α-, and β-glucans. The crude Poli-GL extract had a considerable glucose content (16.6%). However, the partially purified extract, S-Poli-GL, had a high content of total glucans (46.7 ± 3.8%) and β-glucans (43.3 ± 3.1%), and a low content of α-glucans (3.45 ± 0.6%), demonstrating that the freeze–thaw treatment effectively concentrates the glucans. Another study observed similar contents and concluded that *Ganoderma lucidum* is a fungus rich in β-glucans (41.4 ± 1.4%) (Kozarski et al., 2012) [29].

**Figure 2:**
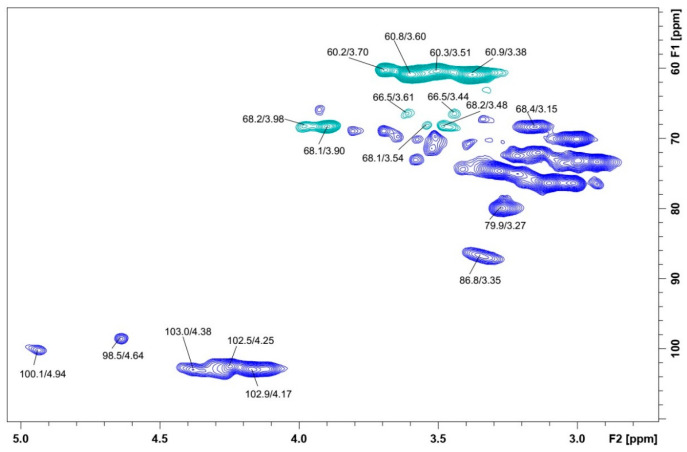
HSQC spectrum of *G. lucidum* extract (S-Poli-GL). The experiment was performed in Me_2_SO-*d*_6_ at 70 °C (chemical shifts are expressed in δ ppm). Blue signals represent positive phase correlations (CH and CH3 groups), while green signals correspond to negative phase correlations CH2 groups).

NMR analysis of S-Poli-GL showed clear signals confirming the presence of β-glucans at high concentrations with small amounts of α-glucans and traces of a galactose-containing polysaccharide (Figure 2). Strong intense signals at δ 103.0/4.38; 102.5/4.25 and 102.9/4.17 in the anomeric region were attributed to β-D-glucose, while two small signals at δ 100.1/4.94 and at δ 98.5/4.64 were attributed to α-D-glucose and α-D-galactose, respectively. The unique and high-intensity signals at δ 86.8/3.35 indicate that most of the binding between glucose units is β-(1⟶3). Furthermore, the β-glucans present in this sample are typically branched at the O-6 position, as confirmed by the inverted signals at δ 68.2/3.48 and 3.98; 68.1/3.54 and 3.90. The intense presence of signals at δ 60.2/3.70 and 3.51; 60.8/3.60 and 3.38 indicates unsubstituted C-6, which are probably part of the β-glucans and part of the α-D-glucans. Low-intensity signals confirming O-6 substitution of α-D-galactose were observed at δ 66.5/3.44 and 3.61.

The results of chemical analysis showed that S-Poli-GL consisted mainly of branched β-glucans with (1⟶3) and (1⟶6) linkages and minor amounts of α-glucans and galactans. This fraction was highly water-soluble and selected for the treatment of animals with mucositis.

### 2.2. Effect of Poli-GL on Weight Loss and DAI

**Figure 3:**
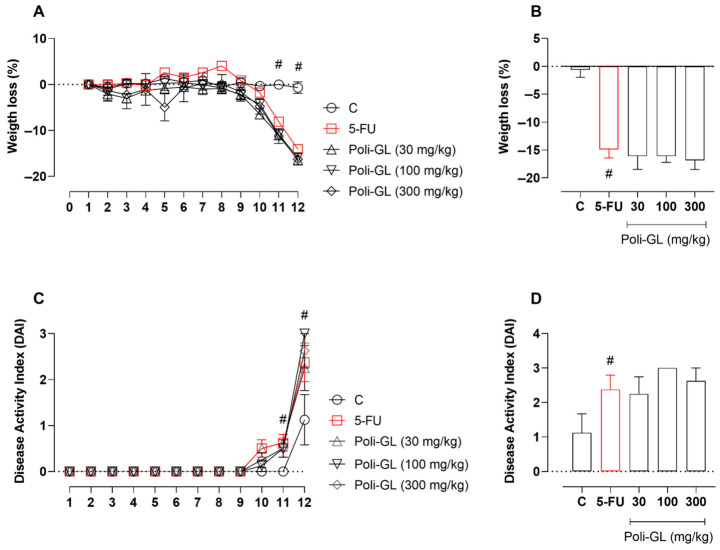
Effect of Poli-GL on (**A**,**B**) body weight changes and (**C**,**D**) disease activity index in 5-fluorouracil (5-FU)-induced intestinal mucositis. Mice received 5-FU to induce mucositis and were treated orally with vehicle (water, 1 mL/kg) or Poli-GL (30, 100, or 300 mg/kg) once daily for the experimental period. Results are expressed as mean ± SEM or median and interquartile range and were analyzed by two-way ANOVA (**A**,**C**) followed by Bonferroni’s post hoc test, or Kruskal–Wallis followed by Dunn’s test (**B**,**D**). # *p* < 0.05 compared to the control group. *n* = 6–8 animals per group.

Despite significant advances in modern oncology, conventional chemotherapeutic agents such as 5-fluorouracil remain widely used in treating various solid tumors (Christensen et al., 2019; Ghafouri-Fard et al., 2021) [30,31]. 5-FU is associated with numerous adverse effects, including inflammation of the intestinal mucosa, tissue ulceration, abdominal pain, and diarrhea, collectively referred to as intestinal mucositis (Dahlgren et al., 2021; Sougiannis et al., 2021) [32,33]. This condition involves epithelial damage, disruption of the mucosal barrier, and activation of inflammatory signaling pathways (Sonis, 2004; Villa et al., 2015) [34,35]. Therefore, developing strategies to prevent or mitigate these toxicities remains a critical need in cancer therapy. In this study, animals treated with 5-FU showed significant weight loss and increased disease activity index (DAI), confirming the establishment of mucositis. However, treatment with Poli-GL did not reverse these parameters (Figure 3). This lack of efficacy suggests that the crude polysaccharide extract may not have adequate structural characteristics or bioavailability to exert protective effects. Similar observations have been reported for non-fractionated mushroom extracts, where biological activity depends highly on polysaccharide composition, molecular weight, and solubility (Zhang et al., 2007; Ferreira et al., 2015) [36,37]. These findings reinforce the importance of extraction and processing strategies in determining the functional properties of *Ganoderma lucidum* polysaccharides.

### 2.3. Effect of S-Poli-GL on Weight Loss and DAI

**Figure 4:**
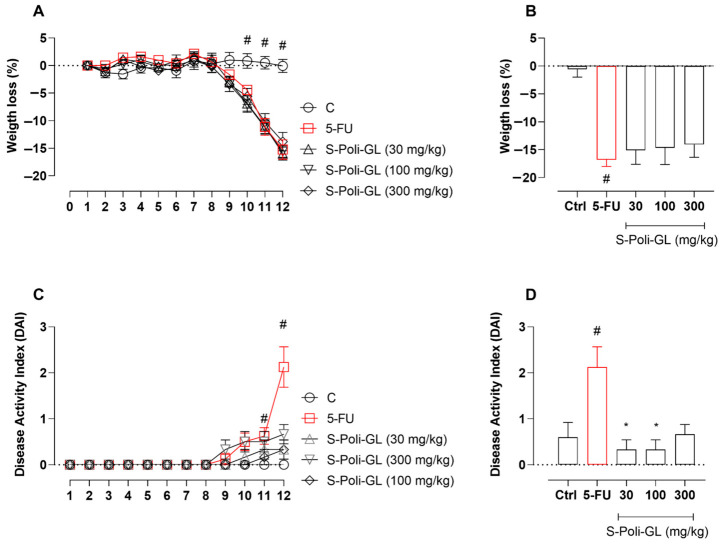
Effect of S-Poli-GL on (**A**,**B**) body weight changes and (**C**,**D**) disease activity index in 5-fluorouracil (5-FU)-induced intestinal mucositis. Mice received 5-FU to induce mucositis and were treated orally with vehicle (water, 1 mL/kg) or S-Poli-GL (30, 100, or 300 mg/kg) once daily for the experimental period. Results are expressed as mean ± SEM or median and interquartile range and were analyzed by two-way ANOVA (**A**,**C**) followed by Bonferroni’s post hoc test, or Kruskal–Wallis followed by Dunn’s test (**B**,**D**). # *p* < 0.05 compared to the control group; * *p* < 0.05 compared to the 5-FU group. *n* = 6–8 animals per group.

Similar to the previous experiment, animals in the 5-FU group lost weight and showed increased DAI. Although treatment with S-Poli-GL did not prevent weight loss, it significantly reduced the increase in DAI (Figure 4), indicating a protective effect on disease severity. β-D-Glucans with (1→3) and (1→6) linkages, such as those present in S-Poli-GL, have been shown to support intestinal health and reduce inflammation triggered by various stimuli (Han et al., 2017; Volman et al., 2008) [38,39]. These polysaccharides exert immunomodulatory effects through interaction with pattern recognition receptors, such as dectin-1, leading to modulation of innate immune responses and cytokine production (Brown & Gordon, 2005; Goodridge et al., 2009) [40,41]. In addition, β-glucans can improve intestinal barrier integrity by enhancing tight junction proteins and mucosal defense mechanisms (Porsani et al., 2017a; Murphy et al., 2020) [42,43].

There is also evidence that β-glucans influence gut microbiota composition, promote beneficial bacterial populations, and increase the production of short-chain fatty acids, which are essential for intestinal homeostasis (Jayachandran et al., 2018) [44]. Although microbiota was not evaluated in this study, these mechanisms may contribute to the protective effects observed. Importantly, to our knowledge, there are no previous studies evaluating *Ganoderma lucidum* polysaccharides in a 5-FU-induced intestinal mucositis model, highlighting the originality and relevance of our findings.

### 2.4. Effect of S-Poli-GL on MPO Activity

**Figure 5:**
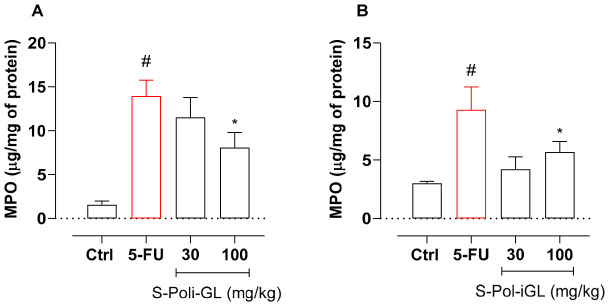
Effect of S-Poli-GL on myeloperoxidase (MPO) activity in 5-fluorouracil (5-FU)-induced intestinal mucositis. (**A**) Duodenum and (**B**) colon. Mice received 5-FU to induce mucositis and were treated orally with vehicle (water, 1 mL/kg) or S-Poli-GL (30, 100, or 300 mg/kg) once daily for the experimental period. Results are expressed as mean ± SEM and were analyzed by one-way ANOVA followed by Bonferroni’s post hoc. # *p* < 0.05 compared to the control group; * *p* < 0.05 compared to the 5-FU group. *n* = 6–8 animals per group.

Intestinal mucositis is characterized by increased oxidative stress, reduced epithelial proliferation, and marked inflammation (Yoneda et al., 2021) [45]. Reactive oxygen species (ROS) production and inflammatory cell infiltration play a central role in disease progression (Basile et al., 2019; Yoneda et al., 2021) [45,46]. Myeloperoxidase (MPO) activity is widely used as an indicator of neutrophil infiltration and tissue inflammation. Consistent with previous studies, 5-FU administration significantly increased MPO activity in both the small intestine and colon, confirming an inflammatory response. Treatment with S-Poli-GL effectively prevented this increase (Figure 5), indicating a strong anti-inflammatory effect. This effect may be related to the ability of β-glucans to regulate immune responses and reduce neutrophil recruitment and activation (Chan et al., 2009; Sevindik, 2025) [47,48]. Additionally, β-glucans have been reported to reduce oxidative stress and modulate inflammatory signaling pathways, helping to preserve intestinal mucosal integrity (Liu et al., 2015; Zhu et al., 2016) [49,50]. Therefore, the reduction in MPO activity observed in this study supports the hypothesis that S-Poli-GL attenuates intestinal inflammation and may protect against mucosal damage induced by chemotherapy.

## 3. Materials and Methods

### 3.1. Reagents

Trifluoroacetic acid (TFA, CAS No. 76-05-1), dimethyl sulfoxide-*d*_6_ (CAS No. 2206-27-1), phosphate-buffered saline (PBS, P4417, MDL MFCD00131855), protease inhibitor cocktail (Sigma FAST™, S8830-20TAB), hexadecyltrimethylammonium bromide (HTAB, CAS No. 57-09-0), and 3,3’,5,5’-tetramethylbenzidine (TMB, CAS No. 207738-08-7) were purchased from Sigma-Aldrich (Burlington, MA, USA). Sodium borohydride (CAS No. 16940-66-2), chloroform (CAS No. 67-66-3), acetic anhydride (CAS No. 108-24-7), pyridine (CAS No. 110-86-1), and hydrogen peroxide (H_2_O_2_, 35% P.A.-A.C.S., CAS No. 7722-84-1) were acquired from Labsynth (Diadema, SP, Brazil). The D-Glucose Assay Kit and the Mushroom and Yeast Beta Glucan Assay Kit were obtained from Megazyme (Wicklow, Ireland). Bovine Serum Albumin (BSA, CAS No. 9048-46-8) was supplied by Inlab (Dortmund, Germany), and sodium acetate (A.C.S., CAS No. 127-09-3) was sourced from Quimibras (Rio de Janeiro, RJ, Brazil). 5-Fluorouracil (Fauldfluor^®^) was provided by Libbs Farmacêutica Ltda. (São Paulo, Brazil). All chemical reagents and solvents used in the experiments were of analytical grade and utilized without further purification unless otherwise stated.

### 3.2. Polysaccharides Isolation and Characterization

#### 3.2.1. Fungal Material

Specimens of *G. lucidum* (strain CC-229) were cultivated in Taboão da Serra (23°36′26″ S and 46°45′07″ W) and provided by Juncao Brazil (Taboão da Serra, SP, Brazil). This specific lineage, originally imported from China, was acquired from the Brazilian Agricultural Research Corporation (Embrapa, Brasília, DF, Brazil). The cultivation substrate (~700 g) consisted of 71.8% eucalyptus sawdust/shavings, 10.2% sugarcane bagasse, 15.4% wheat bran, and 2.6% gypsum, with the addition of 1.4 times the dry weight in water. The substrate was packed into plastic bags for mycelium inoculation. The total cultivation period until harvest lasted approximately 5 months, comprising 30–40 days for substrate colonization and 3–4 months for fruiting body development and sporulation.

#### 3.2.2. Response Surface Methodology (RSM)

Response Surface Methodology (RSM) is a statistical and mathematical approach for fitting a polynomial equation to experimental data based on a selected experimental design. In this study, an experimental design was established to optimize conventional hot-water extraction in an autoclave for recovering *G. lucidum* polysaccharides. A full-factorial three-stage experimental design (3^2^) was selected. The factors analyzed were extraction time (ranging from 20 to 120 min) and ethanol concentration for precipitating the polysaccharides (ranging from 30% to 90%). The response variables were glucose content (an indirect measure of β-glucans) and polysaccharide yield (expressed in grams). Eleven experiments were performed in randomized order, as described in Table 1. The optimal conditions, estimated by multiple linear regression, were recently tested to confirm the empirical results. Statgraphics Centurion XVI software (16.1.15, Statpoint Technologies, Warrenton, VA, USA) was used to design the experiment, analyze the results, and generate the graph.

#### 3.2.3. Polysaccharides Extractions

**Figure 6:**
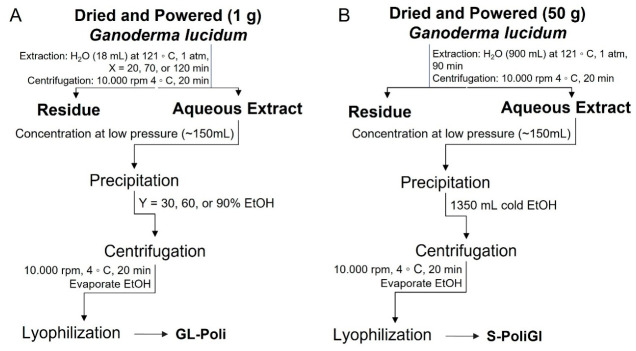
Extraction scheme of polysaccharides from *Ganoderma lucidum* in small (**A**) and medium scale (**B**). X = extraction time (20, 70, or 120 min); Y = ethanol concentration (30%, 60%, or 90%).

Polysaccharide extraction was carried out on a small scale (for the RSM method) and a medium scale (using optimal conditions). The general procedure is shown in Figure 6. Briefly, dried and milled mushroom fruiting bodies (1 or 50 g) were autoclaved with 18 or 900 mL of water for 20–120 min at 121 °C and 1 atm. The extract from each batch was separated from the residue by centrifugation (10,000 rpm) at 4 °C for 20 min. The residue was stored at −20 °C, while the RSM extracts were adjusted to 18 mL with water and precipitated with the appropriate amount of ethanol as described in Table 1. The extract obtained under optimal conditions was concentrated to 150 mL under reduced pressure at 40 °C, followed by the addition of 9 volumes of cold ethanol. Polysaccharides from the RSM extracts and the extract under optimal conditions were recovered by centrifugation (10,000 rpm at 4 °C for 20 min). The remaining ethanol was evaporated using a magnetic stirrer (Dielab Comércio de Produtos Laboratoriais, Curitiba, PR, Brazil) at 60 °C for 2 h before freeze-drying.

*Ganoderma lucidum* was autoclaved, and the extract from each batch was separated from the residue by centrifugation, then concentrated under reduced pressure. The polysaccharide extracts were obtained by ethanol precipitation and lyophilized for further evaluation of chemical properties and in vivo activities.

#### 3.2.4. Determination of Glucan Content

The glucan content in the RSM extracts was determined indirectly by measuring the glucose content as follows: 2 mg of each RSM extract was hydrolyzed with 500 µL of 2 M trifluoroacetic acid (TFA) at 100 °C for 6 h. The acid was then evaporated under a nitrogen flow, and the glucose content was measured using the D-Glucose Assay Kit (GOPOD format) from Megazyme (Wicklow, Ireland) according to the manufacturer’s instructions. The values were expressed as a percentage of glucose (Table 3).

The glucan content in the optimized extract was determined using an enzymatic kit (Mushroom and Yeast Beta Glucan Assay, Megazyme, Wicklow, Ireland) according to the manufacturer’s instructions. Briefly, total glucans were measured by weighing 45 mg of ethanol precipitate into glass tubes, adding 1 mL of 12 M H_2_SO_4_, and placing the tubes on ice for 2 h. Then, 2 mL of H_2_O was added to each tube, and the tubes were incubated in boiling water for 2 h. The hydrolyzed contents were transferred to a 50 mL volumetric flask, mixed with 3 mL of 10 M potassium hydroxide (KOH) solution, and supplemented with 200 mM sodium acetate buffer (pH 5.0). The contents of each flask were mixed by inversion, and 1.5 mL aliquots were centrifuged at 1500× *g* for 10 min. From this, 0.1 mL of each sample was collected and incubated with 0.1 mL of exo-1,3-β-glucanase plus β-glucosidase at 40 °C for 60 min. Then, 3 mL of the glucose determination reagent (GOPOD) was added, and the mixture was incubated at 40 °C for 20 min. Absorbance was measured at 510 nm.

For the measurement of α-glucans, 50 mg of ethanol precipitates were weighed, and 1 mL of 2 M KOH solution was added. The tubes were placed on ice on a magnetic stirrer for 20 min. Then, 4 mL of 1.2 M sodium acetate buffer were added, followed immediately by 0.1 mL of amyloglucosidase plus invertase, and the tubes were incubated at 40 °C for 30 min. For samples containing more than 10% α-glucans, a 0.1 mL aliquot was transferred to a 50 mL volumetric flask, which was filled with distilled water and mixed by swirling several times. Aliquots of 1 mL were centrifuged at 1500× *g* for 10 min. Quantification was performed using the GOPOD reagent as previously described, and absorbance was read at 510 nm. Calculations were performed according to the protocol equations, with appropriate adjustments to the dilutions, and the β-glucan content was determined by subtracting the α-glucan yield from the total glucans.

#### 3.2.5. Polysaccharide Purification

The sample prepared under optimized conditions was named Poli-GL. This sample (0.9 g) was weighed and dissolved in 200 mL of distilled H_2_O for purification by the freeze–thaw method. The solution was kept at 50 °C for 1 h under magnetic stirring to ensure complete dissolution. It was then frozen at −20 °C until completely solidified. The thawing and refreezing process was repeated three times. To separate the supernatant and precipitate, the sample was centrifuged at 10,000 rpm and 4 °C (Rotina 420R Hettich, Tuttlingen, Germany). The supernatant fraction was labeled S-Poli-GL and tested on the animals.

#### 3.2.6. Monosaccharide Analysis

Aliquots (2 mg) of the samples were hydrolyzed with 2 M TFA at 100 °C for 8 h. Each solution was evaporated under nitrogen flow and resuspended in H_2_O (1 mL). Reduction was carried out with NaBH_4_ (2 mg) at room temperature for about 14 h, after which the reduced samples were treated with acetic acid (pH 5.0). The solution was evaporated to dryness under nitrogen flow, and excess boric acid was removed by washing with methanol. Acetylation was carried out with acetic anhydride–pyridine (200 µL; *v*/*v*) at room temperature for approximately 14 h. The resulting alditol acetates were extracted with CHCl_3_ and analyzed by GC–MS (Shimadzu, Kyoto, Japan) using an rtx5-ms column (30 m × 0.25 mm) programmed from 50 to 250 °C at 10 °C/min (Sassaki et al., 2008) [52].

#### 3.2.7. Nuclear Magnetic Resonance Spectroscopy

The NMR experiments (Heteronuclear Single Quantum Correlation—Distortionless Enhancement by Polarization Transfer: HSQC-DEPT) were performed using a 600 MHz Bruker spectrometer model Avance III with a 5 mm inverse probe. The purified fraction (S-Poli-GL) (50 mg) was dissolved in Me_2_SO-*d*_6_, and the analyses were performed at 70 °C. The chemical shifts (δ) were expressed in ppm relative to the solvent resonances ^13^C (δ 39.7) and ^1^H (δ 2.40). NMR signals were assigned based on HSQC-DEPT experiments and literature data.

### 3.3. 5-FU-Induced Gastrointestinal Mucositis

#### 3.3.1. Animals and Ethics Statement

Adult female Balb/C mice (6–8 per group), weighing 20–30 g and aged 4–6 weeks, were obtained from the animal facility of the Carlos Chagas Institute, Fiocruz, Curitiba, PR, Brazil. The animals were housed in groups of up to 12 per cage and acclimatized under controlled conditions: temperature 23 ± 2 °C, humidity 60 ± 10%, and a 12 h light-dark cycle, with ad libitum access to water and standard food. Cages were prepared with wood shavings and environmental enrichment. Bedding was replaced every three days. The animals were acclimated to the researchers before the experiments began. All experimental protocols were approved by the Ethics Committee for the Use of Animals (CEUA) of the Pelé Pequeno Príncipe Research Institute under approval number 076-2024 (approval date: 5 February, 2024).

#### 3.3.2. Experimental Protocol

After the acclimatization phase, the animals were weight-matched and randomly assigned to experimental groups (maximum of 8 per cage): Control group (which received only the vehicle, i.e., water, 0.1 mL/kg, p.o.), the 5-FU group (which received a single intraperitoneal injection of 450 mg/kg 5-FU and was subsequently treated with the vehicle, 0.1 mL/kg, p.o.), and the Poli-GL-treated groups (which received Poli-GL at doses of 30, 100, or 300 mg/kg, p.o., in addition to 450 mg/kg 5-FU).

The animals were pretreated orally once daily for seven consecutive days with either vehicle (water, 0.1 mL/kg) or Poli-GL (30, 100, or 300 mg/kg). On day 8, the mice in the 5-FU and Poli-GL groups received a single intraperitoneal injection of 450 mg/kg 5-FU. Administration of vehicle or Poli-GL continued until day 12. On day 12, all animals were anesthetized with ketamine and xylazine and euthanized.

Shortly thereafter, the purified fraction (S-Poli-GL) was also tested according to the previous protocol: animals were pretreated orally with vehicle (water, 0.1 mL/kg) or S-Poli-GL (30, 100, or 300 mg/kg) daily for seven consecutive days. On day 8, the mice in the 5-FU and S-Poli-GL groups received a single intraperitoneal injection of 450 mg/kg 5-FU. Administration of vehicle or S-Poli-GL continued until day 12. On day 12, all animals were anesthetized with ketamine and xylazine and euthanized.

Organs of interest (duodenum and colon) were removed and preserved in potassium phosphate-buffered solution (PBS, Sigma-Aldrich, St. Louis, MO, USA), pH 7.4, with protease inhibitor (Sigma FAST^TM^, Sigma-Aldrich) for later analysis. The tissue samples were stored at −80 °C until further processing. Throughout the experimental period, the animals were systematically monitored for clinical parameters such as weight loss, stool consistency, and the presence of gross blood in the feces. The severity of diarrhea, an established marker of 5-FU-induced intestinal mucosal damage, was assessed using the disease activity index (DAI) described by Kurita et al. (2000) [51] (Table 3).

#### 3.3.3. Tissue Preparation for the Assessment of Inflammation Parameters

Duodenum and colon samples were homogenized in potassium phosphate-buffered saline (PBS, pH 7.4) with a protease inhibitor cocktail (SigmaFAST™, Sigma-Aldrich, S8830-20TAB) to determine inflammatory markers. The homogenate was centrifuged at 8900 rpm for 20 min at 4 °C. The pellet was resuspended in PBS with hexadecyltrimethylammonium bromide (HTAB, Sigma-Aldrich, CAS No. 57-09-0) and centrifuged again under the same conditions (8900 rpm, 4 °C, 20 min). The supernatant was collected for measurement of myeloperoxidase (MPO), an indirect marker of polymorphonuclear cell infiltration.

#### 3.3.4. Quantification of Proteins

The total protein content in the tissue samples was determined using the Bradford method (Bradford, 1976) [53] and measured at 540 nm. A solution of bovine serum albumin (BSA, Inlab, CAS No. 9048-46-8, Dortmund, Germany) served as the standard, and protein concentrations were calculated by interpolation from the standard curve (Thermo Scientific, QF217385D, Waltham, MA, USA).

#### 3.3.5. Determination of MPO

The samples were resuspended in 80 mM potassium phosphate buffer (pH 5.4) containing hexadecyltrimethylammonium bromide (HTAB, Sigma-Aldrich, CAS No. 57-09-0) and then centrifuged. The resulting supernatant was used to determine myeloperoxidase (MPO) activity, following the protocols described by Bradley et al. (1982) [54].

For MPO quantification, 30 μL of the supernatant was added to 200 μL of a phosphate buffer mixture (0.08 M and 0.22 M) containing 0.017% hydrogen peroxide (H_2_O_2_, Labsynth, 35% P.A.-A.C.S., CAS No. 7722-84-1) in a 96-well plate. The reaction was initiated with 18.4 mM 3,3’,5,5’-tetramethylbenzidine (TMB, Sigma-Aldrich, CAS No. 207738-08-7) and incubated for 3 min at 37 °C. The reaction was stopped with 30 μL of sodium acetate (Quimibras, A.C.S., CAS No. 127-09-3), and the absorbance was measured at 620 nm using a Multiskan SkyHigh Spectrophotometer (Thermo Scientific™, Cat. No. A51119700C). Results were expressed as μg MPO per mg protein.

## 4. Statistical Analysis

The data were analyzed using GraphPad Prism 6 software (GraphPad Software, San Diego, CA, USA). All data sets were first assessed for normality. Parametric data are presented as mean ± standard error of the mean (S.E.M.), and non-parametric data are presented as median and interquartile range. Differences between groups were assessed using one-way or two-way analysis of variance (ANOVA) followed by Bonferroni’s post hoc test, or the Kruskal–Wallis test followed by Dunn’s multiple comparison test. A *p*-value ≤ 0.05 was considered statistically significant.

## 5. Conclusions

This study addressed the need to optimize the extraction and functional characterization of *Ganoderma lucidum* polysaccharides, with a focus on their potential application in chemotherapy-induced intestinal mucositis. Using response surface methodology, we established optimized conditions that enabled efficient recovery of extracts enriched in β-D-glucans. A subsequent fractionation step produced a soluble fraction (S-Poli-GL) predominantly composed of branched β-D-(1→3),(1→6)-glucans, as confirmed by NMR and monosaccharide analyses. Our results demonstrate that the biological activity of *G. lucidum* polysaccharides strongly depends on their structural and physicochemical properties. While the crude extract (Poli-GL) showed limited efficacy, the glucan-enriched soluble fraction significantly reduced disease severity and intestinal inflammation in a 5-fluorouracil-induced mucositis model. To our knowledge, this is the first study to demonstrate the effects of *G. lucidum* polysaccharides in this experimental context.

These findings highlight that optimized extraction and fractionation strategies are critical to enhancing the therapeutic potential of mushroom-derived polysaccharides. Moreover, they support the use of well-characterized β-glucan-rich fractions as promising supportive agents to mitigate chemotherapy-induced intestinal toxicity. Further studies are needed to elucidate the underlying mechanisms and explore their translational potential in clinical settings.

## Figures and Tables

**Table 1:** Full factorial 3^2^ experimental design of *G. lucidum* extractions. Predicted and observed values of each individual response.

	Independent Factors		Investigated Responses	
Run	Time (min)	[EtOH] (%)	Dried Mass (mg)	[Glc] (%)
1	70	60	6.6	15.0
2	20	90	9.9	12.1
3	120	60	6.7	15.0
4	70	90	13.1	12.2
5	120	90	15.0	18.2
6	20	30	4.3	16.6
7	70	60	7.5	14.1
8	120	30	8.3	17.6
9	70	60	7.5	14.4
10	20	60	5.2	11.6
11	70	30	6.7	16.8
Optimized desirability: 0.79		Optimal conditions: 120 min 90% EtOH		
Response		Predicted	Observed	
Dried mass (mg)		15.0	18.8	
[Glc] (%)		15.0	16.6	

**Table 2:** Monosaccharide composition of the extracts obtained from *G. lucidum* in different extraction conditions.

	Monosaccharides (%) ^1^				
Fractions	Ara	Fuc	Man	Glc	Gal
1	-	Tr. ^2^	Tr. ^2^	99.3	Tr. ^2^
2	1.3	Tr. ^2^	1.8	92.0	4.6
3	1.2	-	2.4	88.8	4.6
4	Tr.^2^	-	Tr. ^2^	99.2	Tr. ^2^
5	Tr.^2^	Tr. ^2^	Tr. ^2^	95.0	3.6
6	Tr.^2^	Tr. ^2^	1.1	93.0	5.0
7	Tr.^2^	Tr. ^2^	Tr. ^2^	93.0	4.7
8	Tr.^2^	Tr. ^2^	1.7	94.0	3.0
9	4.6	-	2.2	87.0	4.7
10	1.8	1.4	2.9	88.0	5.6
11	1.3	1.3	1.0	92	4.7

^1^ Alditol acetates obtained on successive hydrolysis, NaBH4 reduction, and acetylation. ^2^ Tr. ≤ 0.9%.

**Table 3:** Scoring system for stool consistency assessment proposed by Kurita et al. [51].

Stool Appearance	Score
Normal	0
Slightly altered or damp	1
Moist with little perianal dirt	2
Moist with perianal dirt	3

## Data Availability

The original contributions presented in this study are included in the article. Further inquiries can be directed to the corresponding author.
